# FUNCTIONAL OUTCOMES IN HOME HEALTH STAY FOR PEOPLE WITH DEMENTIA: INTERSECTIONALITY OF GENDER AND RACE

**DOI:** 10.1093/geroni/igae098.2589

**Published:** 2024-12-31

**Authors:** Rashmita Basu

**Affiliations:** East Carolina University, Greenville, North Carolina, United States

## Abstract

This study explores the difference in functional outcomes of Medicare beneficiaries during a home health (HH) admission across the intersectionality of race and gender because these outcomes may be by virtue of their gender and racial/ethnic composition. This cohort study used data from the 2019 Outcome and Assessment Information Set (OASIS) and Medicare claims (Home Health (HH) and inpatient), enrollment, and Chronic Condition Warehouse (CCW) files from the CMS. Functional outcome was measured as a change in the composite ADL score (grooming, bathing, toileting, dressing, walking, transferring, ambulation, and hygiene) from HH admission to HH discharge. Binary logistic regressions with an outcome variable (1/0: if the composite score was ≤0 (improvement/static) at discharge (1), and 0 if > 0 (decline) during HH admission) were performed. The ADRD cohort was identified based on CCW criteria and ICD-10 codes from inpatient and HH claims. The independent predictor was a six-category intersectionality variable that included White female (reference), White male, Black female, Black female, other female, and other male. The analysis included 81,735 Medicare beneficiaries with ADRD, contributing to 174,093 home health stays. Adjusting for patients’ demographics (age, sex, race/ethnicity), number of diagnoses, caregiving support, and MA enrollment, compared to White females, all the other groups were 3% (95% CI:1.01-1.06) to 18% (95% CI: 1.08-1.26) more likely to have improvement or maintenance of ADL composite score from HH admission to discharge. The combination of gender and race/ethnicity composition is critical in examining the functional outcomes of Medicare beneficiaries using postacute care.

